# For Nocturnal Enuresis

**Published:** 1909-12

**Authors:** 


### For Nocturnal Enuresis.

Belladonna is the best remedy in the treatment of nocturnal enuresis, and children bear it well. Sir William Whitla suggests the following mixture to be prescribed for a child seven years old:

| | | | |
|---|---|---|---|
| ℞ | Potassii bromidi.......... | 20 | (ʒv) |
| | Tinct. belladonna......... | 16 | (ʒiv) |
| | Syr. simplicis............ | 30 | (℥j) |
| | Aq. floris aurantii........ | 120 | (ad. ℥iv) |

M. Sig.: Teaspoonful before retiring.

—*The Practitioner.*

(The parts should always be examined for threadworms, hardened smegma and other external irritants. In young boys the foreskin should be retracted by the obstetrician. Circumcision may be necessary in some cases.—Editor.)

